# Overexpression of miR-128 specifically inhibits the truncated isoform of NTRK3 and upregulates BCL2 in SH-SY5Y neuroblastoma cells

**DOI:** 10.1186/1471-2199-11-95

**Published:** 2010-12-10

**Authors:** Monica Guidi, Margarita Muiños-Gimeno, Birgit Kagerbauer, Eulàlia Martí, Xavier Estivill, Yolanda Espinosa-Parrilla

**Affiliations:** 1 Center for Genomic Regulation (CRG), Genes and Disease Program, Dr. Aiguader 88, 08003 Barcelona, Spain; 2 Public Health and Epidemiology Network Biomedical Research Center (CIBERESP), Barcelona, Spain; 3 Pompeu Fabra University (UPF), Dr. Aiguader 80, 08003 Barcelona, Spain; 4 IBE, Institute of Evolutionary Biology (UPF-CSIC), CEXS-UPF-PRBB, Doctor Aiguader 88, 08003, Barcelona, Spain

## Abstract

**Background:**

Neurotrophins and their receptors are key molecules in the regulation of neuronal differentiation and survival. They mediate the survival of neurons during development and adulthood and are implicated in synaptic plasticity. The human neurotrophin-3 receptor gene *NTRK3 *yields two major isoforms, a full-length kinase-active form and a truncated non-catalytic form, which activates a specific pathway affecting membrane remodeling and cytoskeletal reorganization. The two variants present non-overlapping 3'UTRs, indicating that they might be differentially regulated at the post-transcriptional level. Here, we provide evidence that the two isoforms of *NTRK3 *are targeted by different sets of microRNAs, small non-coding RNAs that play an important regulatory role in the nervous system.

**Results:**

We identify one microRNA (miR-151-3p) that represses the full-length isoform of *NTRK3 *and four microRNAs (miR-128, miR-485-3p, miR-765 and miR-768-5p) that repress the truncated isoform. In particular, we show that the overexpression of miR-128 - a brain enriched miRNA - causes morphological changes in SH-SY5Y neuroblastoma cells similar to those observed using an siRNA specifically directed against truncated *NTRK3*, as well as a significant increase in cell number. Accordingly, transcriptome analysis of cells transfected with miR-128 revealed an alteration of the expression of genes implicated in cytoskeletal organization as well as genes involved in apoptosis, cell survival and proliferation, including the anti-apoptotic factor *BCL2*.

**Conclusions:**

Our results show that the regulation of *NTRK3 *by microRNAs is isoform-specific and suggest that neurotrophin-mediated processes are strongly linked to microRNA-dependent mechanisms. In addition, these findings open new perspectives for the study of the physiological role of miR-128 and its possible involvement in cell death/survival processes.

## Background

Neurotrophins are a family of growth factors that play important roles in the nervous system. They exert multiple functions, being crucial for the survival and maintenance of the central and peripheral nervous system as well as in axon and dendrite patterning. Recent evidence has shown that neurotrophins also act as modulators in synaptic plasticity and are consequently involved in cognitive processes, learning and memory formation [1].

In mammals, the neurotrophin family is composed of four members: nerve growth factor (*NGF*), brain-derived neurotrophic factor (*BDNF*), neurotrophin-3 (*NT3*), and neurotrophin-4/5 (*NT4/5*). Each member binds with high affinity to a specific neurotrophic tyrosine kinase (*NTRK*) receptor: NGF is the preferred ligand for NTRK1 (TrkA), BDNF and NT4/5 for NTRK2 (TrkB), and NT3 for NTRK3 (TrkC) [2]. Upon neurotrophin-induced stimulation, NTRK receptors can activate the Ras/MAPK pathway, the PI3K (phosphoinositide 3-kinase) pathway, and/or PLC-γ1 (phospholipase C gamma 1)-dependent signaling, respectively promoting cell survival, differentiation and activity-dependent plasticity.

Neurotrophins exert neuroprotective activity against different paradigms of neuronal cell death [3-5], linking neurotrophic factors, in particular NGF, to neurodegenerative disorders [6]. Neurotrophins and their receptors have also been implicated in the etiology of psychiatric and mood disorders, often in a dosage-dependent manner [7]. As for many other growth factors, the deregulation of neurotrophin signal transduction is involved in several types of cancers, where NTRK receptor activation can either support or suppress tumor growth. This is the case for example of NTRK3, which is highly expressed in neuroblastomas with good prognosis and highly correlates with patient survival [8].

The human *NTRK3 *gene is located on chromosome 15q25 and spans ~380 kb of genomic DNA (chr15: 86,220,992-86,600,665, March 2006, hg18). It contains 19 introns and undergoes alternative splicing. In humans, three transcript variants have been well characterized: a full-length catalytic form (NM_002530) containing a tyrosine kinase (TK) domain, a full-length isoform (NM_001012338) with an insertion of 14 amino acids in the TK domain - which is less abundant and shows reduced signaling potential [9] - and a single non-catalytic truncated form (NM_001007156) that completely lacks the TK domain [10]. Two major protein isoforms have correspondingly been detected in human brain samples: the full-length, kinase-active 150-kDa receptor (FL-NTRK3) and a truncated non-catalytic isoform (TR-NTRK3) of 50 kDa [11]. The main function that has long been attributed to the truncated isoform is the inhibition of catalytic receptors, which is achieved through a dominant-negative or a ligand-sequestering mechanism. However, a recent work has identified a new signaling pathway activated by the truncated isoform, which links NT3 to downstream molecules affecting membrane remodeling, cytoskeletal reorganization and cell movement, such as the scaffold protein tamalin, the Rac1 GTPase and the adenosine diphosphate-ribosylation factor Arf6 [12].

In humans, the full-length receptor is expressed at low but relatively constant levels throughout development, while the truncated receptor is expressed at moderate levels early in development and increases to reach mature levels by adolescence. In contrast, both full-length and truncated transcripts are uniformly expressed throughout postnatal life and decline in ageing [11]. This discrepancy between changes in protein and mRNA levels of NTRK3 suggests that post-transcriptional regulation may play a role in controlling the expression of the two isoforms, in addition to alternative splicing.

MicroRNAs (miRNAs) are post-transcriptional regulators that have been shown to play a key role in the nervous system. The distribution of miRNAs in the developing and adult nervous system is spatially and/or temporally restricted [13], indicating that they may contribute to the fine-tuning of neuronal gene expression. Structurally, mature miRNAs are small RNAs of approximately 22 nucleotides in length, and regulate gene expression by means of partial complementarity to miRNA binding sites located in the 3' untranslated regions (3'UTRs) of target mRNAs [14]. In particular, perfect complementarity between nucleotide #2 through #7 or #8 at the 5' end of the mature miRNA - the so-called *seed *region - and the 3'UTR of genes is considered determinant for successful binding [15]. Genes with long 3'UTRs are more prone to miRNA-mediated regulation compared with genes with short 3'UTRs, which tend to be specifically deprived of miRNA target sites [16]. Interestingly, full-length and truncated *NTRK3 *transcripts show different 3'UTR regions that do not overlap, supporting the hypothesis that the two isoforms may be differentially regulated by miRNAs. Furthermore, it has recently been shown that miRNAs miR-9 and miR-125a/b are able to specifically regulate the expression of the truncated isoform of the NTRK3 receptor [17].

Only one study has so far analyzed the contribution of miRNAs to determining the balance between different variants of the same gene [18]. Here, we show that the full-length and truncated isoforms of *NTRK3 *are regulated by different sets of miRNAs. In addition, we demonstrate that, among the miRNAs that inhibit the truncated isoform, the overexpression of miR-128 - a brain-enriched miRNA - in SH-SY5Y neuroblastoma cells alters the expression profile of genes involved in cytoskeletal organization and of genes related with apoptosis and cell cycle regulation, including the anti-apoptotic factor BCL2.

## Methods

### Firefly luciferase constructs

The 3'UTRs of the full-length and truncated isoforms of *NTRK3 *were PCR-amplified from BAC CTD-2508H23 with *PfuTurbo*^® ^DNA polymerase (Stratagene, La Jolla, CA), using primers containing an *Xba*I restriction site at the 5' end: forward 5'-acacactctagagtctgccccaaagaggtgta-3' and reverse 5'-acacactctagaccaaactgccttacagggttt-3' for the full-length isoform and forward 5'-acacactctagaaataagccttcccggacatt-3' and reverse 5'-acacactctagatgcaaaatttccaaataagagg-3' for the truncated isoform. PCR fragments of 334 and 2110 bp respectively were purified, *Xba*I-digested and cloned into an *Xba*I site located downstream of the firefly luciferase reporter gene in the pGL4.13 vector (Promega Corporation, Madison, WI). Constructs were propagated in *E. coli *One Shot^® ^TOP 10 cells (Invitrogen). Mutant reporter plasmids were generated as previously described [19] using the QuikChange site-directed mutagenesis kit (Stratagene, La Jolla, CA, USA), with the wild-type pGL4.13 construct as a template and primers carrying the desired point mutations. The authenticity and orientation of the inserts as well as the presence of the mutations were confirmed by direct sequencing.

### Cell culture and transfection

HeLa cells and SH-SY5Y neuroblastoma cells were maintained in Dulbecco's Modified Eagle's Medium supplemented with 10% fetal bovine serum, 2 mM L-glutamine, 100 units/ml penicillin and 100 μg/ml Streptomycin (GIBCO™, Invitrogen). In the case of SH-SY5Y cells, fetal bovine serum was heat inactivated for 45 min at 56°C prior to use. Transfection with small RNAs was performed with Lipofectamine 2000 (Invitrogen), using either small RNAs that mimic endogenous mature miRNAs and the related negative controls (miRIDIAN™miRNA Mimics and miRIDIAN™miRNA Mimics Negative Controls #2 and #4, Dharmacon Inc.) or miRNA inhibitors and the corresponding negative control (miRCURY LNA™ microRNA Inhibitors and miRCURY LNA™ microRNA Inhibitor Negative Control A, Exiqon). The cotransfection of plasmid DNA and small RNAs into HeLa cells was optimized using a GFP-containing plasmid (pEGFP-C1, Clontech) and a commercially available GFP-specific siRNA (Ambion), obtaining a transfection efficiency of over 80%. Transfection of SH-SY5Y cells with miRNA mimics was optimized and monitored in each experiment using a fluorescein-labeled double-stranded RNA oligomer (siGLO Green Transfection Indicator, Dharmacon). Optimal efficiency was obtained at a concentration of 50-200 nM and miRNA mimics as well as miRNA inhibitors were ultimately used at 100 nM.

### Luciferase activity assay

HeLa cells were seeded at 1.3 × 10^4 ^cells/well in 96-well plates and cotransfected 24 h later with the Firefly reporter constructs described above or the empty pGL4.13 vector (10-24 ng), the Renilla reporter plasmid pGL4.75 (3 ng) and the appropriate miRNA mimic (30 nM). The activity of Firefly and Renilla luciferases was determined 24 h after transfection using the Dual-Glo™ Luciferase Assay System (Promega). Relative reporter activity was obtained by normalization to the Renilla luciferase activity. In order to correct for vector-dependent unspecific effects, each relative reporter activity was normalized to the empty vector cotransfected with the corresponding miRNA. Results were then compared to the mean of the two negative controls. Each experiment was done in triplicate and at least three independent experiments were performed for each miRNA. Statistical significance was determined using Student's *t *test (p < 0.05).

### Western blotting

For the analysis of NTRK3 expression during RA-induced differentiation, SH-SY5Y cells were treated with 10 μM *all-trans*-RA and harvested at time 0 (untreated), day 3, day 6 and day 10 of RA treatment. For TR-NTRK3, undifferentiated SH-SY5Y cells were plated at 2 × 10^5 ^cells per well in 6-well plates and transfected 24 h later with 100 nM miRNA mimic; 72 h after transfection cells were lysed and analyzed by western blotting. For FL-NTRK3, SH-SY5Y cells were differentiated with 10 μM *all-trans*-RA, plated in 6-well plates at a concentration of 3 × 10^5 ^cells per well at day 3 of RA treatment, transfected with 100 nM miRNA mimic at day 4 of RA treatment and analyzed 72 h after transfection (day 7 of RA treatment).

For protein extraction, cells were rapidly rinsed with ice-cold PBS and solubilized with RIPA buffer: 50 mM Tris-HCl pH 7.4, 150 mM NaCl, 2 mM EDTA, 0.1% SDS, 1% Nonidet P 40, 1% sodium deoxycholate, 1 mM Na_3_VO_4_, 1 mM PMSF, 50 mM NaF and 1× protease inhibitors (Complete Mini, EDTA-free, Roche). Cells were then scraped off, incubated on ice for 15 min and centrifuged at 12000 rpm for 15 min. Samples were resolved in NuPAGE^® ^4-12% Bis-Tris polycrylamide gels using the NuPAGE^® ^MES SDS Running Buffer and transferred to nitrocellulose membranes using the iBlot™Dry Blotting System (Invitogen). Before blotting, gels were equilibrated in 100 ml equilibration buffer (2× NuPAGE^® ^Transfer Buffer, 10% methanol and 1:1000 NuPAGE^® ^Antioxidant) for 20 min at room temperature (RT; all NuPAGE^® ^products are from Invitrogen).

Immunodetection was performed using the ODYSSEY^® ^infrared imaging system (LI-COR^® ^Biosciences), following the manufacturer's instructions for two-color western blotting. This detection system allows the precise quantification of low-abundance proteins (as is the case of TR-NTRK3) for which chemiluminescence is not enough sensitive and accurate, and has the advantage that the signal generated by the proteins on the membrane is measured in a static state. Membranes were blocked in ODYSSEY^® ^blocking buffer for 1 h at RT and incubated with the appropriate primary antibody (Ab) for 1 h at RT (anti-TrkC goat polyclonal IgG, Upstate, catalog number 07-226; anti-Bcl-2 (N-19), Santa Cruz, catalog number sc-492; anti-Caspase-3, Millipore, catalog number 06-735; anti-Caspase-9, Cell Signaling, catalog number 9508). As a loading control, membranes were simultaneously incubated with an anti-GAPDH Ab (anti-GAPDH mouse monoclonal Ab, Chemicon, catalog number MAB374). Blots were subsequently probed with the appropriate fluorophore-labeled secondary antibodies (LI-COR^® ^Biosciences) and finally scanned on an ODYSSEY^® ^infrared scanner. Fluorescent bands were quantified using the ODYSSEY^® ^software. Each experiment was repeated at least four times; data are reported as means ± SE and statistical significance was determined using Student's *t *test.

For the analysis of ERK phosphorylation, differentiated cells were seeded and transfected as explained above; 72 h after transfection cells were rinsed twice with serum-free medium, incubated with serum-free medium for 5 h and then acutely stimulated for 10 min with 30 ng/mL neurotrophin-3 (Alomone Labs). Cells were then lysed in 50 mM Tris-HCl pH 7.4, 50 mM NaF, 10 mM sodium pyrophosphate and 0.005% Triton X-100. After transfer, membranes were incubated with an anti-phopsho-ERK (Anti-MAP kinase, activated mouse monoclonal Ab, Sigma, catalog #M9692) and an anti-pan-ERK Ab (Anti-p44/42 MAP kinase rabbit polyclonal Ab, Cell Signaling, catalog # 9102). To make sure that the quantification was accurate in each WB, a gradient (5, 10 and 15 μg) of one of the control samples was loaded on each gel. The gradients of FL-NTRK3, TR-NTRK3 and GAPDH were quantified by densitometry, and the corresponding standard curve equations were calculated for each WB; blots were considered reliable only if standard curves showed a correlation coefficient (R^2^) > 0.9 (Additional file 1). Standard curve equations were then applied to the densitometry counts of each sample for quantification.

### miRNA expression analysis using custom microarrays (Agilent, 11k)

Total RNA, including short RNAs, was extracted from HeLa and SH-SY5Y cells using the miRNeasy^® ^Mini Kit (QIAGEN). miRNA expression in HeLa and SH-SY5Y cells was analyzed using custom 11k oligonucleotide microarrays (Agilent Technologies), including probes for the 325 known human miRNAs according to Sanger miRBase release 7.1. The expression values obtained were compared with results reported in other studies [20,21], and the general correlation was good; however, to avoid possible false results due to a failure in the hybridization of specific individual probes, we eliminated from subsequent analysis those miRNAs whose expression values were discordant with the other studies. Negative controls included probes for 20 *Bacillus subtilis*-specific sequences and 20 rare non-human sequences. Two μg of total RNA were labeled with Hy5™or Hy3™fluorescent labels using the miRCURY™LNA microRNA Labelling kit (Exiqon), following the manufacturer's instructions. Pairs of labeled samples were hybridized to dual-channel microarrays for 40 h at 55°C using Agilent hybridization reagents. Microarray images were quantified using the GenePix 6.0 (Axon) software; only spots with signal intensities twice above the local background and not saturated were considered reliable and used for subsequent analysis. Extracted intensities were subtracted from the local background and the log_2 _ratios were normalized in an intensity-dependent fashion. Statistical analyses were performed using the MMARGE tool.

### Whole-genome expression analysis using beadchip microarrays

Total RNA samples obtained from four independent experiments (SH-SY5Y cells transfected with miR-128 and the related negative controls) were analyzed on HumanRef-8 BeadChips from Illumina, which target 24,500 well-annotated RefSeq transcripts. Starting from 200 ng of total RNA, biotin-labeled cRNA was synthesized and hybridized according to the manufacturer's instructions. Data were analyzed using the Array File Maker (AFM) 4.0 software package. The whole genome microarray data obtained here are part of a larger project that is still ongoing. These results will be deposited in MIAME format in the ArrayExpress public repository once the experiment is completed.

### Real-time quantitative RT-PCR

DNase-treated RNA extracted from transfected cells was retrotranscribed with the SuperScript™III First-Strand Synthesis System for RT-PCR (Invitrogen). RNA levels of the full-length and truncated isoforms of *NTRK3 *were analyzed by real-time quantitative RT-PCR using SYBR Green I (Roche), following the manufacturer's instructions. Reactions were performed with the LightCycler^® ^480 Instrument (Roche) in 384-multiwell plates. Specific primers were designed for the two *NTRK3 *isoforms (FL-NTRK3 forward 5'-cagccatggttccaactctc-3', FL-NTRK3 reverse 5'-ccagcatgacatcgtacacc-3', TR-NTRK3 forward 5'-tccagagtggggaagtgtct-3', TR-NTRK3 reverse 5'-ccatggttaagaggcttgga-3') and for *GAPDH *(forward 5'-ctgacttcaacagcgacacc-3', reverse 5'-ccctgttgctgtagccaaat-3'), which was used as housekeeping gene. All amplicons were ~100 bp in length, and forward and reverse primers were located in different exons. Thermal cycling was performed as follows: one pre-incubation cycle at 95°C for 10 min (ramp rate: 4.8°C/s); 45 amplification cycles at 95°C for 10 sec, 57°C for 30 sec and 72°C for 5 sec (ramp rates: 4.8, 2.2 and 4.8°C/s). The expression levels of miR-128 in different human tissues and in SH-SY5Y cells were analyzed using the TaqMan^® ^MicroRNA Assays, following the manufacturer's instructions.

### Cell counting

Cells cultured on 6-well plates and transfected with miR-128, the TR-NTRK3-specific siRNA (sense 5'-gagucuaugccuuuggcaatt-3', antisense 5'-uugccaaaggcauagacuctt-3', Gene Link) and the related negative controls were trypsinized and resuspended in 1 mL DMEM; 100 μL of each sample were then diluted in 10 mL of Coulter Isoton II diluent (Beclman Coulter) and counted using a Z2™ Series Coulter Particle Count and Size Analyzer (Beckman Coulter).

### Flow cytometry

SH-SY5Y cells transfected with miR-128 and the related negative controls were trypsinized and resuspended in PBS 72 h after transfection cells. Samples were examined using a BD FACSCanto Flow Cytometer and the BD FACSDiva software (BD Biosciences). A two-parameter forward/side scatter (FSC/SSC) analysis was performed recording 5000 events in each analysis.

### Computational methods

Three web-based miRNA target prediction methods were used: miRanda (http://www.microrna.org, 2005 release; [22]), TargetScan (http://www.targetscan.org, releases 2.0, 2.1, 3.0 and 3.1; [15]) and PicTar (http://pictar.mdc-berlin.de; [23]). Genomic coordinates were according to the human assembly release of March 2006 (H. sapiens hg18). Sequences were obtained from the University of California Santa Cruz (UCSC) Genome Browser http://www.genome.ucsc.edu. Sequence analysis was performed using the 4peaks software http://mekentosj.com/4peaks/ and Multalin http://multalin.toulouse.inra.fr/multalin/. Primers were designed using the Primer3 software http://frodo.wi.mit.edu/cgi-bin/primer3/primer3.cgi and PCR annealing temperatures were calculated with the Optimase ProtocolWriter™ http://mutationdiscovery.com. Pathway analysis from microarray expression data was performed with the Ingenuity Pathway Analysis Software (IPA) version 6.3 http://www.ingenuity.com.

## Results

### Functional screening of miRNA target sites in NTRK3

The full-length and the truncated isoform of *NTRK3 *were searched for putative target sites with three widely used miRNA target prediction programs - miRanda, TargetScan and PicTar. Taking into account the 325 human miRNAs annotated in the miRbase database at the time of the analysis (version 7.1, October 2005) we could identify 3 miRNAs predicted to target the full-length isoform and 29 miRNAs predicted to target the truncated isoform (Table 1).

**Table 1:** Tested miRNAs and predicted target

miRNA	FL-NTRK3	TR-NTRK3
hsa-let-7b†		M(1), T(1)
hsa-let-7e†	M(1)	
hsa-miR-1	NP	NP
hsa-miR-9#		T(1)
hsa-miR-10a	NP	NP
hsa-miR-15a	NP	NP
hsa-miR-16	NP	NP
hsa-miR-17-3p	NP	NP
hsa-miR-17-5p†		T(2)
hsa-miR-18a*		M(1)
hsa-miR-18a*	NP	NP
hsa-miR-20a†		T(2)
hsa-miR-24#		T(1)
hsa-miR-30e-3p	NP	NP
hsa-miR-30e-5p	NP	NP
hsa-miR-93†		T(1)
hsa-miR-103	NP	NP
hsa-miR-106a†		T(2)
hsa-miR-106b†		T(2)
hsa-miR-107	NP	NP
hsa-miR-125a§		M(2), T(2)
hsa-miR-125b		M(2)
hsa-miR-128†		M(1), T(1)
hsa-miR-133a#		M(1)
hsa-miR-141	NP	NP
hsa-miR-149#		M(1), T(1)
hsa-miR-151-3p	M(1)	
hsa-miR-182	NP	NP
hsa-miR-185	M(1), P(1)	
hsa-miR-188		M(1)
hsa-miR-198		M(1)
hsa-miR-200a	NP	NP
hsa-miR-200b	NP	NP
hsa-miR-204#		M(1), T(1)
hsa-miR-206	NP	NP
hsa-miR-211#		M(1), T(1)
hsa-miR-221	NP	NP
hsa-miR-296		M (1)
hsa-miR-324-5p§		M(1), T(1)
hsa-miR-326		M(2)
hsa-miR-330-3p¥		M(2), T(2)
hsa-miR-331		M(2)
hsa-miR-340		M(1)
hsa-miR-345¥		T(1)
hsa-miR-374	NP	NP
hsa-miR-384§		T(1)
hsa-miR-412		M(1)
hsa-miR-422a#		M(1), T(1)
hsa-miR-449		M(1)
hsa-miR-485-3p§		T(1)
hsa-miR-509¥		M(1), T(2)
hsa-miR-617¥		T(1)
hsa-miR-625¥		T(2)
hsa-miR-765¥		T(1)
hsa-miR-768-5p¥		T(1)

M, miRanda; T, TargetScan; P, Pictar; NP, not

predicted for either isoform; † and #, conserved

sites and poorly conserved sites for miRNA families broadly

conserved among vertebrates, respectively; §, poorly

conserved sites for miRNA families conserved only among mammals;

¥, poorly conserved sites or poorly conserved miRNA families.

The number of predicted sites is indicated in brackets.

The entire 3'UTRs of the full-length and truncated isoforms of *NTRK3 *(178 and 1981 bp, respectively) were cloned immediately downstream of the firefly luciferase open reading frame in the pGL4.13 plasmid, and the resulting constructs were designated pGL4.13-FL and pGL4.13-TR. The renilla reporter plasmid pGL4.75 was used to control for transfection efficiency. A total of 55 miRNAs and 2 non-targeting controls were tested on both pGL4.13-FL and pGL4.13-TR. Tested miRNAs (Table 1) included the 32 mentioned above; 6 additional miRNAs that appeared in more recent versions of the miRbase database and had putative target sites in the truncated isoform (miR-485-3p, miR-509, miR-617, miR-625, miR-765 and miR-768-5p) were also included because allelic variants were found in their target sites after re-sequencing of patients with anxiety disorders [19]. Eleven out of the 35 miRNAs were predicted to regulate the truncated isoform by more than one method, and at least 7 were conserved target sites for miRNA families broadly conserved among vertebrates (Table 1). Finally, 17 miRNAs with no predicted target sites according to the interrogated methods were also analyzed for comparison.

**Figure 1:**
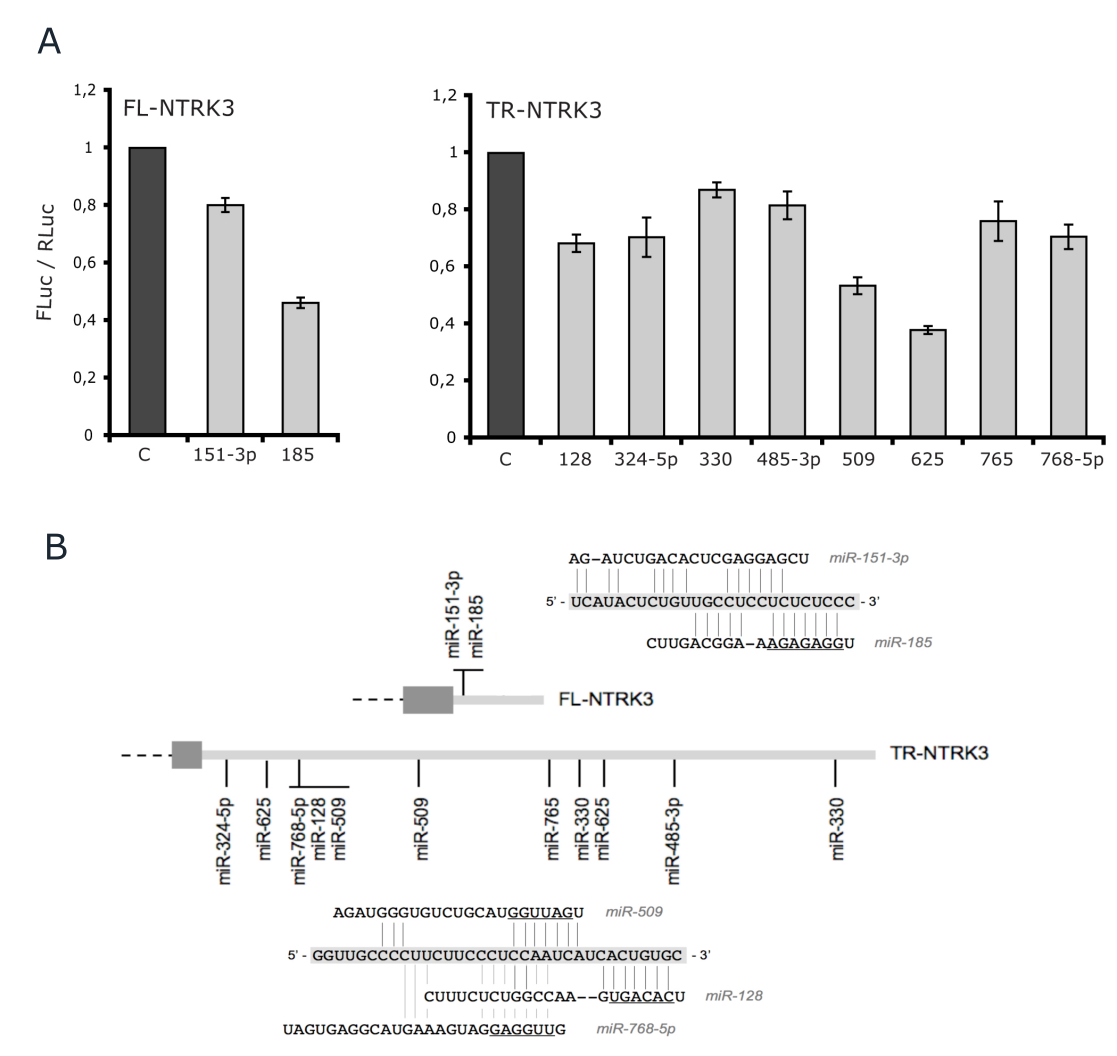
**Functional screening of NTRK3 miRNA target sites using a luciferase-based assay**. A. Luciferase assay results: FL-NTRK3 and TR-NTRK3 regulation by miRNAs. HeLa cells were cotransfected with pGL4.13-FL or pGL4.13-TR and the indicated miRNA mimics (C = Control). Luciferase activities were measured 24 h after transfection; firefly luciferase activity was normalized to Renilla luciferase activity, and results from at least three independent experiments are presented as means ± SE. Only statistically significant results are shown (p < 0.05). B. The relative location of the target sequences of validated miRNAs in the 3'UTRs of FL-NTRK3 and TR-NTRK3 is shown with an enlargement of overlapping target sites, where sequence pairing to the 3'UTRs (lines) and miRNA seed regions (underlines) are indicated.

The specific luciferase activity of the pGL4.13-FL construct was significantly decreased by 2 miRNAs predicted to target the full-length isoform (miR-151-3p and miR-185, Figure 1A). The strongest repression was observed with miR-185 (54% reduction), followed by miR-151-3p (20%). In the case of pGL4.13-TR, the luciferase activity was significantly reduced by 8 miRNAs (Figure 1A), all of which were predicted by at least one program: miR-128, miR-324-5p, miR-330, miR-485-3p, miR-509, miR-625, miR-765 and miR-768-5p. The most conspicuous inhibition was detected with miR-625 (62% reduction), miR-509 (47%) and miR-128 (32%), while the other five miRNAs gave a reduction ranging between 13% and 30%.

Since multiple target sites in the same 3'UTR are thought to be cooperative and to increase the efficacy of miRNA inhibition, we analyzed whether the cotransfection of different miRNAs repressing pGL4-13-TR could lead to a synergistic effect and a more dramatic reduction in luciferase activity. Thirteen combinations of two miRNAs were tested, but we could not detect a significant decrease in luciferase expression compared with the corresponding miRNAs taken individually; a combination of the three most effective miRNAs - miR-128, 509 and 625 - was also analyzed and no synergistic effect was observed (Additional file 2).

### miRNA-mediated regulation of endogenous NTRK3 in neuroblastoma cells

SH-SY5Y neuroblastoma cells were used to investigate whether miRNAs causing a significant decrease in luciferase activity were also able to downregulate endogenous NTRK3. Upon exposure to retinoic acid (RA), SH-SY5Y cells reduce their growth rate and differentiate by extending neurites into cells that are biochemically, ultrastructurally and electrophysiologically similar to neurons [24]. Furthermore, RA treatment has been shown to induce the appearance of functional FL-NTRK3 receptors in this cell line [25].

**Figure 2:**
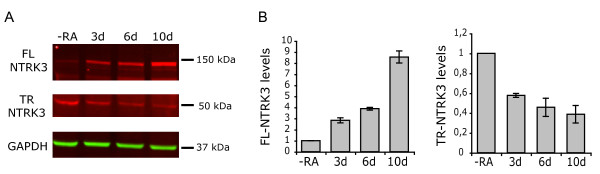
**Characterization of the expression of NTRK3 in RA-differentiated SH-SY5Y cells**. A. Immunoblotting of the full-length and truncated isoforms of NTRK3 in SH-SY5Y cells treated with RA for the indicated times. GAPDH was used as a loading control. B. Protein levels were quantified by densitometry and are reported as arbitrary units relative to untreated cells.

We characterized the expression of NTRK3 in SH-SY5Y cells at different time points during RA exposure by western blotting. As previously described for another neuroblastoma cell line [17], we could observe an increase in the levels of the full-length isoform and a decrease in the truncated isoform. Full-length protein levels were ~2.5-fold higher after three days of RA treatment, ~4-fold higher after six days and reached up to ~8-fold after ten days. On the other hand, the expression of the truncated isoform was reduced by approximately 50% after three days of exposure, and kept decreasing very slightly through day 10 (Figure 2). Given these expression profiles, untreated cells were used to study the regulation of the truncated isoform, and RA-differentiated cells were used for the full-length isoform.

**Figure 3:**
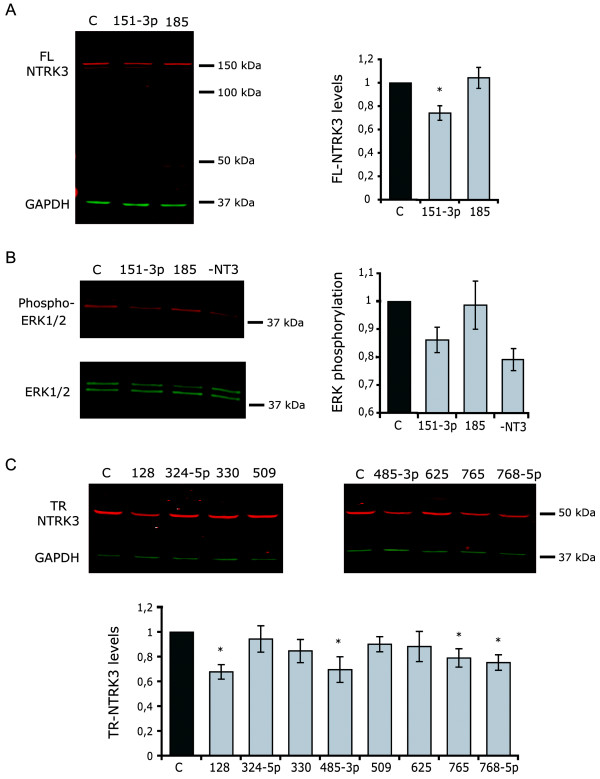
**Analysis of miRNA-mediated regulation of endogenous NTRK3 in neuroblastoma cells**. A. Western blot analysis of NTRK3 levels in RA-differentiated cells transfected with mimics targeting FL-NTRK3; NTRK3 levels are reported relative to control cells. B. Analysis of ERK1/2 phosphorylation after acute stimulation of differentiated SH-SY5Y cells with NT3. Basal phosphorylation in unstimulated cells is also shown (-NT3). C. NTRK3 levels in undifferentiated cells transfected with mimics targeting TR-NTRK3. Data from four independent experiments are presented as means ± SE (*p < 0.05).

The endogenous expression of 7 luciferase-validated miRNAs was also analyzed in SH-SY5Y cells, using custom oligonucleotide microarrays (Table 2). The expression of these miRNAs was in general very low, making miRNA overexpression, rather than the use of miRNA antagonists, the method of choice to analyze the regulation of endogenous NTRK3 in this cell system. Luciferase-validated miRNAs were therefore transfected into either undifferentiated (miR-128, miR-324-5p, miR-330, miR-485-3p, miR-509, miR-625, miR-765 and miR-768-5p) or differentiated SH-SY5Y cells (miR-151-3p and miR-185), and protein levels were assessed by western blotting 72 h after transfection. In agreement with the luciferase assay data, FL-NTRK3 levels were significantly reduced by miR-151-3p (34%); a slight inhibition was also observed with miR-185, but did not reach statistical significance (Figure 3A). To investigate whether such inhibition of FL-NTRK3 was able to affect the efficiency of NT3-induced signaling, we analyzed ERK1/2 phosphorylation by acutely stimulating transfected cells with NT3 after serum starvation. ERK phosphorylation was used as a readout for the activation of the Ras/MAPK pathway, which is triggered by stimulation of full-length, kinase-active NTRK receptors [26]. We could detect a reduction in ERK phosphorylation with miR-151-3p but not with miR-185 (Figure 3B), which is consistent with the reduction observed in the levels of FL-NTRK3 after overexpression of miR-151-3p.

**Table 2:** miRNA expression levels in HeLa and SH-SY5Y cells

miRNA	HeLa	Untreated SH-SY5Y	Differentiated SH-SY5Y (RA 6 days)
**miR-128**	2.3	5.1	7.5
**miR-151-3p**	1.5	3.9	4.8
**miR-185**	3.7	4.6	4.7
**miR-324-5p**	1.3	1.1	1.6
**miR-330**	1.8	6.2	6.7
**miR-485-3p**	1.1	7.9	7.9
**miR-509**	1.1	3.3	3.3

RA, retinoic acid. Expression levels are indicated relative to background signal.

**Figure 4:**
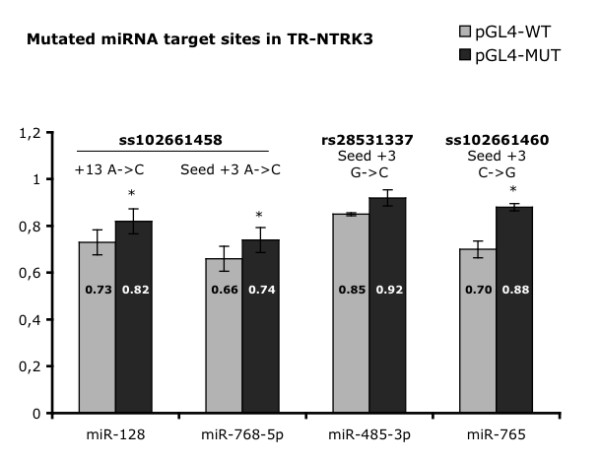
**Effect of miRNA overexpression on allelic variants at the 3'UTR of TR-NTRK3**. Luciferase activity of wild-type (pGL4-WT) and mutated (pGL4-MUT) TR-NTRK3 constructs corresponding to allelic variants ss102661458, rs28521337 and ss102661460 cotransfected with the indicated miRNA mimics. Results from at least three independent experiments are presented as means ± SD (*p < 0.05).

As for TR-NTRK3 (Figure 3C), a significant downregulation, ranging between 20% and 30%, was observed with miR-128, miR-485-3p, miR-765 and miR-768-5p; the strongest repression was caused by miR-128 (32% reduction) and miR-485-3p (30%). Three natural mutations occurring in the 3'UTR of TR-NTRK3 had been previously identified, which fall within the predicted binding sites of these four miRNAs [19]: ss102661458 at the binding sites for miR-768-5p (A-> C at the +3 seed region position) and miR-128 (+13 position), rs28521337 at the binding site for miR-485-3p (G-> C at the +3 seed region), and ss102661460 at the binding site for miR-768-5p (C-> G at the +3 seed region). We generated pGL4.13-TR point mutants that resemble these naturally occurring mutations and could observe a significant recovery of luciferase activity in mutant constructs (Figure 4), which supports the predicted direct miRNA-mRNA binding interactions. The remaining four miRNAs (miR-324-5p, miR-330-3p, miR-509 and miR-625) caused a slight reduction in the expression levels of the truncated isoform of NTRK3 (maximum 15%), but none of them reached statistical significance. In the case of miR-128, the miRNA that caused the strongest reduction in TR-NTRK3 levels, we performed antisense experiments using LNA miRNA inhibitors. Blocking endogenous miR-128, we could observe a slight increase (over 10%) in the levels of TR-NTRK3 compared with the control (Additional file 3); however, the difference did not reach statistical significance, probably due to the low basal expression of miR-128 in this cell system.

Finally, full-length and truncated *NTRK3 *transcripts were quantified by real-time quantitative RT-PCR after miRNA overexpression, using isoform-specific primers. mRNA levels were not affected by any of the regulating miRNAs, indicating that they do not act by destabilizing *NTRK3 *transcripts and suggesting that the observed downregulation is achieved through translational repression.

### miR-128 overexpression affects the morphology and number of SH-SY5Y cells

**Figure 5:**
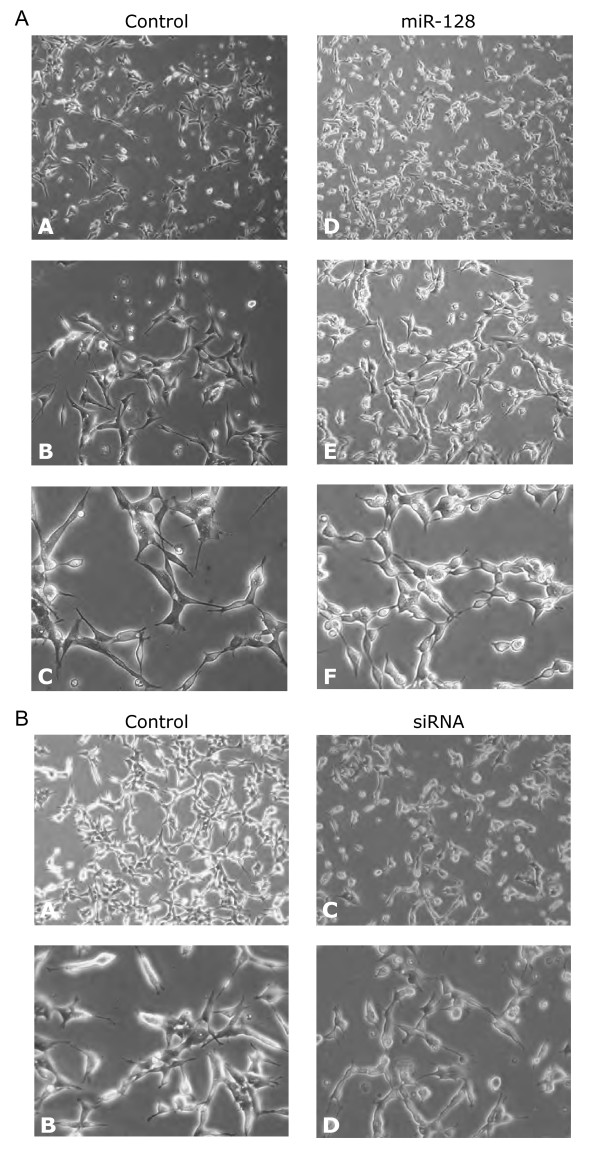
**Phase-contrast micrographs of undifferentiated SH-SY5Y cells**. A. Cells were transfected with a non-targeting control (A,B,C) and with miR-128 (D,E,F); morphological changes (rounded bodies, shorter neurites and smaller cell size) were observed in cells transfected with miR-128. B. Cells were transfected with a non-silencing control (A,B) and with an siRNA directed against the truncated isoform of NTRK3 (C,D). The morphology of cells transfected with the siRNA resembles that of cells transfected with miR-128.

**Figure 6:**
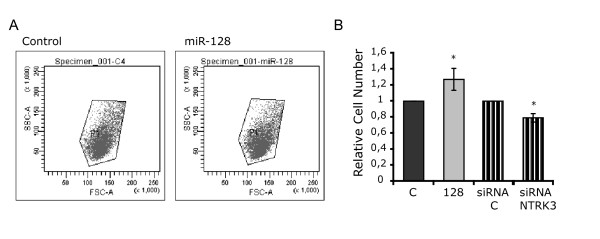
**Characterization of cells transfected with miR-128 and a TR-NTRK3-specific siRNA**. A. Two-parameter forward/side scatter (FSC/SSC) flow cytometry analysis of SH-SY5Y cells transfected with a non-targeting control and miR-128. No profile shift was observed. B. Counting of undifferentiated SH-SY5Y cells transfected with miR-128, the a TR-NTRK3-specific siRNA and the corresponding negative controls. Results from three independent experiments are reported as means ± SE, relative to the respective controls (*p < 0.05).

After transfection with miRNA mimics, cells were examined under a phase-contrast microscope to check for possible alterations induced by miRNA overexpression. While in most cases there were no appreciable differences, considerable changes were observed after transfection with miR-128 (Figure 5A): cells acquired rounded bodies with shorter neurites, the overall cell size looked smaller than control cells and the culture confluence appeared to be higher, suggesting an increase in the total number of cells. Given that miR-128 downregulates TR-NTRK3, it was reasonable to speculate that the repression of this variant could be responsible for at least part of the observed effects. Cells were therefore transfected with an siRNA directed against TR-NTRK3, which targets an isoform-specific sequence located within the 3'UTR region and reduces TR-NTRK3 levels by approximately 25% - a degree of repression comparable to that observed with miR-128. Interestingly, the morphology of cells was similar to that described for miR-128 (Figure 5B), supporting the hypothesis that TR-NTRK3 plays a part in the morphological phenotype. In order to characterize these changes, two-parameter forward/side scatter (FSC/SSC) flow cytometry was performed, revealing no shift in the population of cells transfected with miR-128 (Figure 6A). This indicates that there is no variation in the actual size or cytoplasmic complexity of cells and that the observed alterations are due to other factors, possibly involving a modification of the adhesive properties or in the motility of the cell. Finally, transfected cells were counted with a Coulter cell counter, showing that the total number of cells in plates transfected with miR-128 was 27% higher than in control plates (Figure 6B).

**Figure 7:**
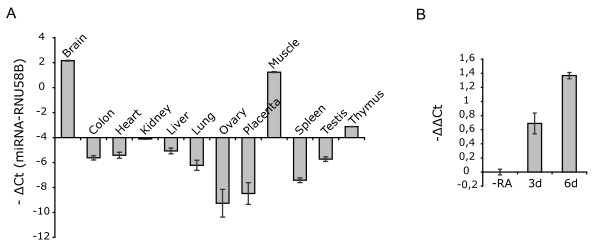
**Real-time RT-PCR analysis of miR-128 expression using TaqMan^® ^miRNA assays**. A. Results for different human tissues are reported as -ΔCts between each miRNA and the endogenous control (small nucleolar RNA RNU58B). ΔCt = 0 corresponds to the expression level of RNU58B in each sample. B. Results for SH-SY5Y cells during RA-mediated differentiation are reported as -ΔΔCts, relative to untreated cells (reference sample: -RA).

miR-128 is a brain-enriched miRNA, whose expression has been shown to correlate and increase with neuronal differentiation [27,28]. The expression of miR-128 was analyzed by real-time quantitative RT-PCR in a set of human tissues (adult brain, colon, heart, kidney, liver, lung, ovary, skeletal muscle, spleen, testis, thymus and placenta) as well as in SH-SY5Y cells at different stages of RA treatment. The analysis confirmed that miR-128 is strongly expressed in the brain, and high levels were also detected in skeletal muscle, followed by thymus and kidney (Figure 7A). In SH-SY5Y cells, in agreement with the miRNA microarray experiment described before, miR-128 showed low levels of expression, with average crossing point (Ct) values ranging from ~33 to ~35. Nevertheless, we could indeed observe an increase in miR-128 expression upon RA treatment (Figure 7B). This change in miR-128 levels, also observed by microarray analysis, is consistent with the hypothesis that it contributes to the repression of TR-NTRK3 during RA-mediated differentiation of SH-SY5Y cells.

### Transcriptome analysis of SH-SY5Y cells transfected with miR-128

In order to gain insight into the role of miR-128, the effects of its overexpression were further analyzed using whole genome expression microarrays (Illumina's HumanRef-8 v3.0 beadchips). Considering a fold-change (FC) cutoff of 1.2 and a q-value <5, we could identify a total of 183 deregulated genes after miR-128 overexpression in SH-SY5Y cells - 116 downregulated and 67 upregulated - with a maximum FC of ~1.8 for upregulated genes and -2 for downregulated genes. The top ten upregulated and downregulated genes are listed in Tables 3 and 4, respectively. Interestingly, among them are several genes implicated in apoptosis, cell death/survival and proliferation, with a general tendency for those leading to cell death to be downregulated and for those leading to survival to be upregulated. The most striking example is *BCL2 *(FC = 1.69), a well-known antiapoptotic gene that inhibits caspase activity. The upregulation of *BCL2 *in miR-128-transfected cells could explain the observed increase in cell number, which is consistent with enhanced apoptosis inhibition. Also interesting is *PAIP2 *(FC = -2.03), a translational repressor that inhibits the Vascular Endothelial Growth Factor (*VEGF*), a potent mitogen and survival factor with neuroprotective functions in the brain. The deregulation of other genes like *NGFRAP1*, *PRSS3*, *TRAIF3IP2, CREB3L2 *and of the proapoptotic factors *TXNIP *(Thioredoxin Interacting Protein, FC = -1.38) and *DAP3 *(Death Associated Protein 3, FC = -1.36) follows the same trend. Other strongly deregulated genes are involved in vesicular transport, like *DYNC1I1*, and in cytoskeletal organization like *TMSB10*, *TRAPPC4 *and *VBP1 *(Table 3 and 4). This is in accordance with the observed morphological phenotype and with the involvement of TR-NTRK3 in membrane remodelling, actin reorganization and cell movement [12].

**Table 3:** Top 10 upregulated genes upon miR-128 overexpression in SH-SY5Y cells

GENE	FC			FUNCTION
***DYNC1I1***		1.781		Microtubule-based vesicular trafficking;retrograde axonal transport of signaling endosomes containing neurotrophins and associated downstream kinases
				
***HMGCS1***		1.728		Lipogenesis and biosynthesis of cholesterol
				
***BCL2***		1.689		Regulation of cell death and caspase activity; antiapoptotic
				
***CREB3L2***		1.652		Transcription factor of the CREB3 family; astrocyte proliferation in response to inflammation and trauma (gliosis)
				
***TRAF3IP2***		1.558		Activation of the transcription factor NFkB; implicated in malignant processes and in the regulation of the apoptotic response; mediates the antiapoptotic effect of TGF-beta and TNF-alpha
				
***ISLR2***		1.544		Integral membrane protein
				
***KLF6***		1.544		Zinc finger transcription factor; adipogenesis and inhibition of cellular growth
				
***PRICKLE1***		1.508		May regulate neurite formation during brain development
				
***ADIPOR1***		1.504		Component of the energy homeostatic mechanism; increases fatty acid oxidation and glucose uptake; may participate in the control higher brain functions
				
***PRSS3***		1.498		Encodes mesotrypsin, a brain-specific trypsin form thought to protect neural cells from apoptotic cell death

**Table 4:** Top 10 downregulated genes upon miR-128

GENE		FC		FUNCTION
***TMSB10***		-1.583		Organization of the cytoskeleton, cell motility and spreading: binds to actin monomers and inhibits actin polymerization; anti-apoptotic action in chickembryo motoneurons
				
***TRAPPC4***		-1.589		Vesicular transport from endoplasmic reticulum to golgi; possible role in postsynaptic membrane trafficking
				
***G6PC3***		-1.595		Regulation of glucose homeostasis: catalyzes hydrolysis of G6P to glucose and phosphate
				
***VPB1***		-1.617		Chaperone protein; maturation of the cytoskeleton and morphogenesis; possible role in the transport and correct localization of the tumor suppressor von Hippel-Lindau (VHL) protein
				
***NGFRAP1***		-1.637		p75NTR-associated protein; mediates apoptosis in response to NGF
				
***PKIA***		-1.699		PKA inhibition
				
***YWHAB***		-1.713		Member of the brain-abundant 14-3-3 protein family; may play a role in linking mitogenic signaling and the cell cycle machinery
				
***PAIP2***		-2.034		Translational repressor; acts on VEGF (Vascular Endothelial Growth Factor)
				
***CHGB***		-2.050		Secretory protein expressed in neuronal cells; guides and sorts the secretion of neuropeptides
				
***TROVE2***		-2.069		RNA-binding protein; major autoantigen in patients suffering from autoimmune diseases; binds small RNAs of unknown function (Y RNAs) and misfolded noncoding RNAs; possible function in the degradation of defective RNAs

**Figure 8:**
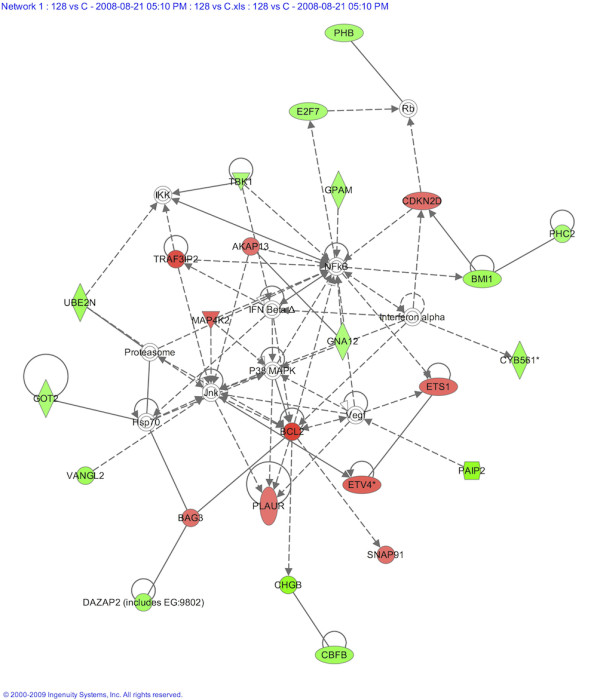
**Molecular network showing the highest percentage of genes altered by the overexpression of miR-128**. Downregulated and upregulated genes are shown green and red, respectively. The analysis was performed using the Ingenuity Pathway Analysis software.

The 183 genes deregulated upon miR-128 overexpression, with the corresponding expression values, were uploaded into the Ingenuity Pathway Analysis software, and the program was interrogated about biological functions, canonical pathways and molecular networks that could be affected. We found associations with biological functions such as cell cycle, cancer, neurological disease and cell death (-log(p-value) >3) and with metabolic canonical pathways (-log(p-value) >3). Enzymes implicated in these pathways show a marked predominance of downregulation over upregulation, indicating that metabolic processes may in general be inhibited or impaired in miR-128-transfected cells. Interestingly, the molecular network showing the highest percentage of deregulated genes has *BCL2 *as one of its central nodes and is associated with cancer, gene expression and neurological disease (Network 1, Figure 8).

Finally, microarray results were compared to target predictions, in order to check whether any of the deregulated genes could potentially be direct targets of miR-128; such genes would undergo miRNA-mediated regulation through mRNA cleavage rather than translational repression. Table 5 shows the intersection of the 183 genes deregulated by miR-128 overexpression (FC cutoff of 1.2 and a q-value <5) and the putative target genes predicted by TargetScan. Of 669 total predicted miR-128 targets, 38 were deregulated (5.7%), a number that is higher than expected by chance (chi square test, p < 10^-6^) and indicates a significant enrichment for deregulated genes among predicted targets. As expectable, there was a huge predominance of downregulation over upregulation, with just one upregulated gene out of 38.

**Table 5:** Target predictions and genes deregulated by miR-128

GENE	FC	GENE	FC
***TROVE2***	-2.07	**PHB**	-1.27
***PAIP2***	-2.03	***PPAP2B***	-1.26
***YWHAB***	-1.71	***ATP6V1A***	-1.25
***PKIA***	-1.70	***STAG1***	-1.21
***NGFRAP1***	-1.64	***PRKX***	-1.21
***C1orf144***	-1.62	***RPS6KB1***	-1.16
***NGFRAP1***	-1.61	***CABLES2***	-1.14
***TMSB10***	-1.58	***EIF2S2***	-1.13
***UBE2E2***	-1.58	***RGL2***	-1.10
***SEC61A1***	-1.56	SH3BGRL2*	1.34
***GLTP***	-1.53	***DAZAP2***	-1.43
***VANGL2***	-1.52	***FBXO33***	-1.43
***C12orf34***	-1.51	***VPS4B***	-1.43
***UBE2N***	-1.50	***PDHX***	-1.41
***CASC3***	-1.49	***SLC39A11***	-1.41
***DCUN1D4***	-1.48	***ZZZ3***	-1.30
***WDR68***	-1.46	***E2F7***	-1.28
***RNF144***	-1.46	***GPAM***	-1.28
***PPP1CC***	-1.44	***PDIA5***	-1.28

Overlapping between target predictions for miR-128 (TargetScan) and genes deregulated by its overexperession. *Only one out of 38 deregulated and predicted target genes was upregulated

### Overexpression of miR-128 upregulates BCL2

**Figure 9:**
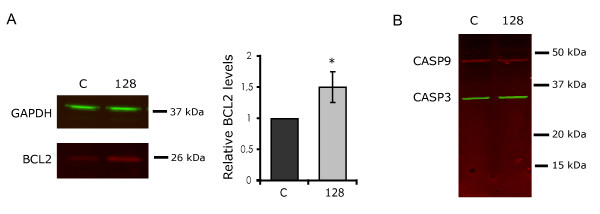
**Western blot analysis of BCL2 levels and caspase activation in undifferentiated cells transfected with miR-128**. A. BCL2 levels show a ~1.5-fold increase relative to the negative control (three independent experiments, *p < 0.05). B. Analysis of caspase-3 (green) and caspase-9 (red) activation reveals that neither caspase is present in its activated form (~17 KDa and ~35 KDa, respectively).

*BCL2 *has no predicted target sites for miR-128 and, as previously mentioned, resulted to be upregulated upon miR-128 overexpression (FC = 1.69, third most upregulated gene) in our microarray experiment. Such upregulation was validated by western blotting, which showed an increase of approximately 1.5-fold at the protein level (Figure 9A) - similar to that observed by microarray for the transcript. The possible effect of the upregulation of BCL2 on the activation of apoptotic markers, such as caspase-3 and caspase-9, in cells transfected with miR-128 was also analyzed by western blotting. As shown in Figure 9B, the active forms of both caspases are practically undetectable, indicating that the basal level of apoptosis in SH-SY5Y cells is already low; as a consequence, it was not possible to appreciate any decrease in the activation of the two caspases. However, although better known as an antiapoptotic factor, BCL2 also plays a role in the regulation of the cell cycle, which appears to be at least in part separate from its antiapoptotic function. It has been shown that BCL2 has an antiproliferative effect, driving cells into enhanced G0 arrest. Enhanced G0 could possibly explain the increase in cell number associated with miR-128 and is compatible with the general downregulation of metabolic pathways indicated by the microarray analysis. MTT assays performed on SH-SY5Y cells revealed no change in total mitochondrial activity after transfection with miR-128 (data not shown) despite the larger number of cells, again in agreement with a general reduction of metabolic activity, which is typical of the quiescent state.

## Discussion

In this study we explore how miRNAs take part in the regulation of two isoforms of the neurotrophin-3 receptor *NTRK3*, which present non-overlapping 3'UTRs. We demonstrate that the two isoforms are targeted by different sets of miRNAs, providing new evidence for a role of miRNAs in determining the balance among different splice variants of a gene. In fact, we identify one miRNAs regulating the full-length isoform of *NTRK3 *(miR-151-3p) and 4 miRNAs regulating the truncated isoform (miR-128, miR-485-3p, miR-765 and miR-768-5p).

Our approach followed two steps: a first screening of miRNAs with predicted targets in the 3'UTR of either isoform, performed in HeLa cells using luciferase constructs, and a subsequent validation of the ability of luciferase-positive miRNAs to repress endogenous NTRK3 receptors in SH-SY5Y neuroblastoma cells. Upon exposure to RA, SH-SY5Y differentiate into cells that are similar to neurons [24]; the presence of FL-NTRK3 receptors after RA stimulation had previously been demonstrated in this cell system [25], where we could well characterize the expression of both NTRK3 isoforms.

If we compare the results obtained with the two strategies, 5 out of 10 (50%) luciferase-positive miRNAs were validated on endogenous NTRK3. Given that the constitutive expression of the remaining 5 miRNAs is similarly low in both HeLa and SH-SY5Y cells, we could rule out the possibility that high levels of endogenous miRNAs in SH-SY5Y cells affected the results of the validation, making the overexpression of miRNA mimics less effective; such rate of false-positives is probably related with the artificial context in which the 3'UTRs are inserted in the luciferase assay strategy.

One limitation of this study is that, due to the generally low expression levels of the analyzed miRNAs in SH-SY5Y cells, the experiments were based on miRNA overexpression, rather than inhibition of endogenous miRNAs. It would be certainly interesting to analyze the effect of miRNA inhibition in a different cell system where the miRNAs of interest are highly expressed, including cell lines where NTRK3 is not present and where the inhibition of the regulating miRNAs might indeed induce its expression. In addition, the levels of the truncated isoform of NTRK3 were also low in SH-SY5Y, and standard WB quantification by enhanced chemiluminescence detection proved to be not enough sensitive. We therefore used fluorescent detection, employing fluorescently-labelled secondary antibodies and an infrared imaging system, which allows the precise quantification of low-abundance proteins [29], and has the advantage that the signal generated by the proteins on the membrane is measured in a static state, as opposed to the enzymatic reaction of chemiluminescence, which is dynamic and changes over time [30]. Given that the signal with this detection system is linear and not exponential as in chemiluminescence, the differences between bands are less apparent but the quantification by densitometry becomes much more accurate. This allowed us to detect the subtle changes originated by the overexpression of miRNAs.

Interestingly, miR-151-3p and miR-185 have partially overlapping target sequences in the full-length isoform and, in a similar way, miR-128, miR-509 and miR-768-5p target the same segment of the 3'UTR of the truncated isoform (Figure 1B). It is worth of notice that while both miR-128 and miR-509 cause a strong reduction in luciferase activity (30-50%), only miR-128 seems to repress the corresponding protein isoform in SH-SY5Y cells. Intriguingly, miR-128 is expressed in brain, whereas miR-509 is not present in brain but shows a strong expression in kidney and testis. This suggests that the binding of these miRNAs to their target sequences might be mutually exclusive, and that the regulation of *NTRK3 *by miRNAs could be coordinated in a tissue specific fashion.

The second part of this study focused on miR-128, one of the miRNAs repressing the truncated isoform. miR-128 is a brain-enriched miRNA, whose expression has been shown to positively correlate with neuronal differentiation [27,28]. Furthermore, accumulation of miR-128 has been detected in the hippocampus of Alzheimer's disease brains [31]. Here, we show that the overexpression of miR-128 causes morphological changes in SH-SY5Y cells, which resemble those observed using an siRNA specifically directed against TR-NTRK3. In accordance with the morphological changes, as revealed by microarray analysis, miR-128 alters the expression of genes involved in cytoskeletal organization, a process with which the truncated isoform has been related. These results suggest an involvement of miR-128 in the organization of the cytoskeleton through the regulation of NTRK3, possibly in cooperation with other miR-128 targets.

Another consequence of the overexpression of miR-128 in SH-SY5Y cells is an increase in cell number, which is accompanied by the deregulation of genes involved in apoptosis, cell death/survival and proliferation, with a remarkable upregulation of BCL2. *BCL2 *codes for an outer mitochondrial membrane protein that blocks cytochrome *c *release from mitochondria and inhibits caspase activity, suppressing apoptosis. However, the analysis of caspase-3 and caspase-9 activation revealed that in normal culture conditions there is virtually no apoptosis in SH-SY5Y cells, making the hypothesis of an increased inhibition of this process unsuitable for our results. It would be therefore interesting to analyze the effects of miR-128 in the presence of apoptosis-promoting agents.

Besides apoptosis, BCL2 also plays a role in the regulation of the cell cycle, having an antiproliferative effect that drives cells into enhanced G0 arrest. This is thought to promote cell survival, especially in unfavorable environments, since quiescent cells are more resistant to killing than proliferating cells [32]. Although this could be could be compatible with the increase in cell number and with the general reduction of metabolic activity observed in our analyses, further investigation is needed to support this hypothesis and to exhaustively evaluate the contribution of *BCL2 *to the observed phenotype, as well as that of other proliferation-regulating genes whose expression is altered by miR-128.

## Conclusions

The full-length and truncated isoforms of *NTRK3 *are regulated by different sets of miRNAs, demonstrating that the regulation of *NTRK3 *by microRNAs is isoform-specific and indicating that neurotrophin-mediated processes are strongly linked to microRNA-dependent mechanisms. In addition, overexpression of the brain-enriched miRNA miR-128 - one of the miRNAs that regulates the truncated isoform of *NTRK3 *- causes morphological changes in neuroblastoma cells and alters the expression profile of genes involved in cytoskeletal organization, apoptosis and cell proliferation, including the anti-apoptotic factor BCL2. These findings open new perspectives for the study of the physiological role of miR-128 and its possible involvement in cell death/survival processes.

## Authors' contributions

Y.E.P. and X.E. conceived and coordinated the study. M.G. and Y.E.P designed research. E.M. and M.M.G helped with the design of some experiments. M.M.G helped in the *in silico *analysis of miRNA target sites. M.G. and B.K. performed the experiments. M.G. and Y.E.P. analyzed data and wrote the paper. All authors revised and approved the final manuscript.

## Supplementary Material

Additional file 1 **WB of a protein gradient (A) with the corresponding standard curves for TR-NTRK3 (B) and GAPDH (C)**. 5, 10 and 15 μg of a control sample were loaded on each protein gel and standard curves were calculated for each immunoblot. Click here for file

Additional file 2 **Analysis of miRNA synergism in TR-NTRK3 using a luciferase based assay**. HeLa cells were cotransfected with pGL4.13-TR and the indicated combinations of miRNA mimics (X axis, C = Control). Luciferase activities were measured 24 h after transfection; firefly luciferase activity was normalized to Renilla luciferase activity, and results from at least three independent experiments are presented as means ± SE. Click here for file

Additional file 3 **Representative WB experiment of SH-SY5Y cells transfected with an anti-miR-128 LNA inhibitor and a control**. Although an increase in the levels of TR-NTRK3 was observed with the anti-miR-128, the difference did not reach statistical significance (three independent experiments were performed). Click here for file
