# Supplementary material for: Overexpression of miR-128 specifically inhibits the truncated isoform of NTRK3 and upregulates BCL2 in SH-SY5Y neuroblastoma cells
Source: BMC Mol Biol. 2010 Dec 10;11:95. doi: 10.1186/1471-2199-11-95 (PMC3019150; doi:10.1186/1471-2199-11-95)
Supplement: Additional file 1 — WB of a protein gradient (A) with the corresponding standard curves for TR-NTRK3 (B) and GAPDH (C). 5, 10 and 15 μg of a control sample were loaded on each protein gel and standard curves were calculated for each immunoblot. [file 1471-2199-11-95-S1.PDF]

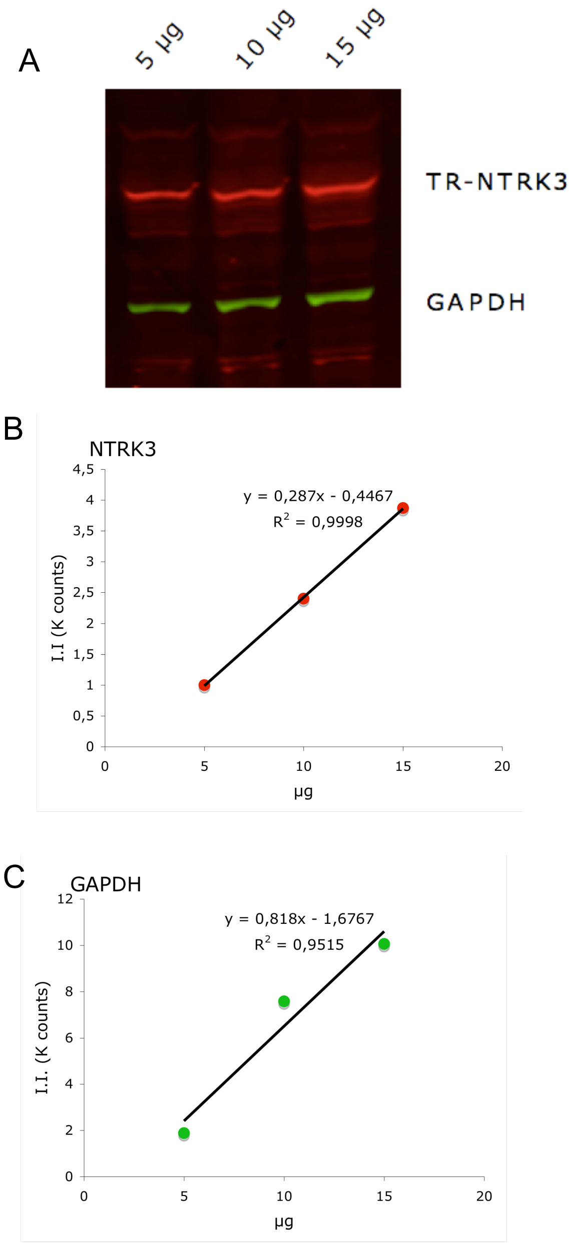

Additional file 1. WB of a protein gradient (A) with the corresponding standard curves for TR-NTRK3 (B) and GAPDH (C). 5, 10 and 15 µg of a control sample were loaded on each protein gel and standard curves were calculated for each immunoblot. **.**
