# Supplementary material for: Overexpression of miR-128 specifically inhibits the truncated isoform of NTRK3 and upregulates BCL2 in SH-SY5Y neuroblastoma cells
Source: BMC Mol Biol. 2010 Dec 10;11:95. doi: 10.1186/1471-2199-11-95 (PMC3019150; doi:10.1186/1471-2199-11-95)
Supplement: Additional file 2 — Analysis of miRNA synergism in TR-NTRK3 using a luciferase based assay. HeLa cells were cotransfected with pGL4.13-TR and the indicated combinations of miRNA mimics (X axis, C = Control). Luciferase activities were measured 24 h after transfection; firefly luciferase activity was normalized to Renilla luciferase activity, and results from at least three independent experiments are presented as means ± SE. [file 1471-2199-11-95-S2.PDF]

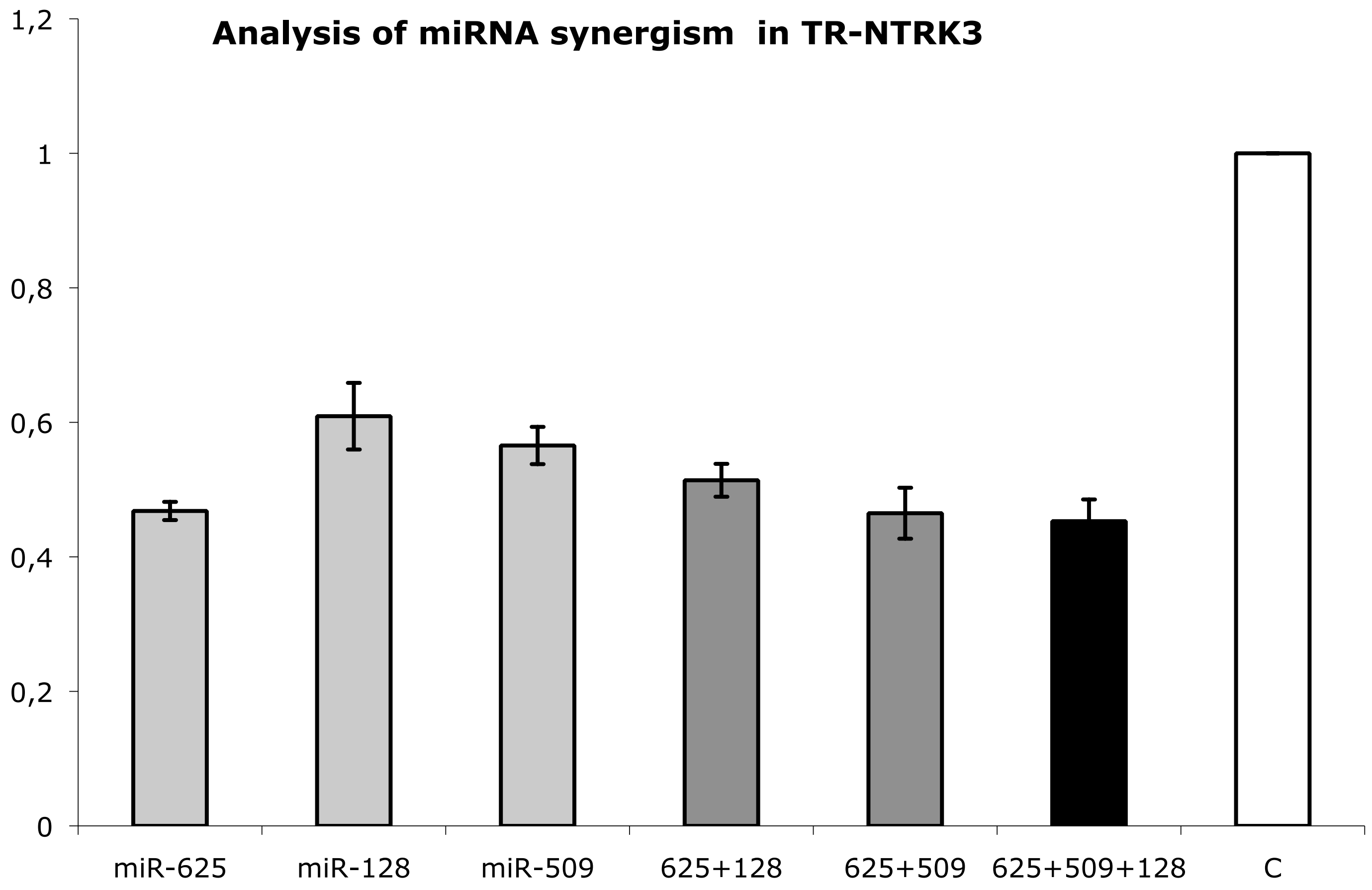

Additional file 2. Analysis of miRNA synergism in TR-NTRK3 using a luciferase based assay. HeLa cells were cotransfected with pGL4.13-TR and the indicated combinations of miRNA mimics (X axis, C=Control). Luciferase activities were measured 24 h after transfection; firefly luciferase activity was normalized to Renilla luciferase activity, and results from at least three independent experiments are presented as means ± SE.
