# Supplementary material for: Overexpression of miR-128 specifically inhibits the truncated isoform of NTRK3 and upregulates BCL2 in SH-SY5Y neuroblastoma cells
Source: BMC Mol Biol. 2010 Dec 10;11:95. doi: 10.1186/1471-2199-11-95 (PMC3019150; doi:10.1186/1471-2199-11-95)
Supplement: Additional file 3 — Representative WB experiment of SH-SY5Y cells transfected with an anti-miR-128 LNA inhibitor and a control. Although an increase in the levels of TR-NTRK3 was observed with the anti-miR-128, the difference did not reach statistical significance (three independent experiments were performed). [file 1471-2199-11-95-S3.PDF]

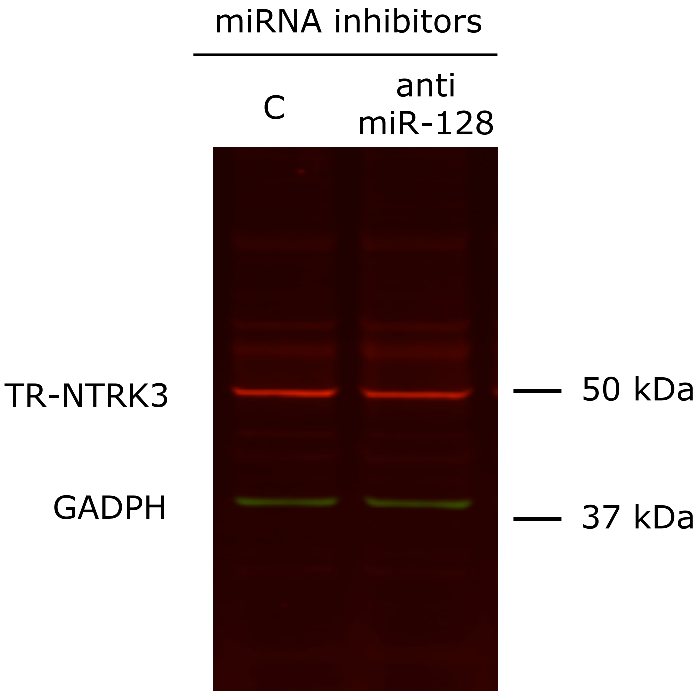

	**TR-NTRK3**	**GADPH**	**TR-NTRK3/ GADPH**
C	7.79	4.07	**1.91**
miR-128	10.25	4.31	**2.37**

Additional file 3. Representative WB experiment of SH-SY5Y cells transfected with an anti-miR-128 LNA inhibitor and a control. Although an increase in the levels of TR-NTRK3 was observed with the anti-miR-128, the difference did not reach statistical (three independent experiments were performed).
